# Institutional Notes and News

**Published:** 1910-10-29

**Authors:** 


### Institutional Notes and News.

**Aberdeen Hospital for Sick Children.**

Recognising that the present building was a makeshift from the first, and is now "hopelessly out of date," the directors of the Royal Aberdeen Hospital for Sick Children are appealing for funds to provide a larger building on a new site as a memorial to King Edward. They support their proposal by pointing out that the hospital has now been working for thirty-four years, and the number of children treated has risen steadily until now, and that with its present tale of only eighty cots the institution is unable to meet the demands made upon it. They propose that the new building should contain at least a hundred beds, and its cost is expected to be from £30,000 to £35,000. The fact that not Aberdeen only, but North-East Scotland, and, indeed, so remote a quarter as the islands of Orkney and Shetland, send patients to the hospital is an argument in favour of extension the force of which is obvious. The new site has not yet been decided on, for it must be not merely adequate in size and healthy in situation, but also near enough to the University of Aberdeen for the hospital to continue to be a teaching centre.

**Macclesfield General Infirmary.**

The opening of the renovated men's ward at the Macclesfield Infirmary, while proving the alert interest of the authorities, brings forward by contrast the remainder of the building, which has now been built for forty years. The work done already in the ward abovementioned has cost about £850, and in order to renovate the entire building some £2,500 would be needed. This sum the governors are hoping to raise, and certainly the renovated ward is practical testimony to the care with which money is spent. A terrano floor, laid upon the existing wooden one, duresco-treated walls, heating by radiation, and ventilating window-hoppers, a hot-air faience stove—these are samples of the renovation which has accompanied the introduction of the fourteen new beds, ten of which are of an adjustable pattern. Mr. F. F. Grafton, C.C., the honorary secretary, is to be congratulated on the results of his enterprise, for he and Mr. Hanrahan, the secretary, have done a large share of the hard work. The matron, Miss L. Drewitt, will appreciate the new ward, and she may genuinely do so, for she seconded the secretaries' efforts in every way.

**Leicester Infirmary.**

At the recent quarterly meeting of the governors of the Leicester Infirmary, Sir Edward Wood, J.P., presided over a big attendance, the large recreation-room being well filled. The top ward of the new wing (the "Gertrude Rogers" ward), named after Miss Rogers, who for twenty-six years had served the institution most faithfully as lady superintendent, has now been opened for female surgical cases. The number of beds in the institution has now reached 265. During the past nine months the number of in-patients treated has been 2,581—an increase of 161 on the corresponding period of last year. In the out-patients' department the daily attendance has averaged 141, with 9,935 new cases, and 25,524 renewed attendances have been recorded—a total of 35,459. It is interesting to note there have been 921 attendances in the *x*-ray department. The balcony system has been so much appreciated by the medical and surgical staff that capital will have to be provided later to extend the system to other wards. As regards the income of the institution for the past nine months, all items have shown an increase except legacies, which, amounting to £1,085, as against £2,199, were less by one-half. The expenditure has increased by £692, the higher prices of most commodities largely accounting for this. The larger number of in-patients is also a contributory cause. There remains a great need for additional income, although annual subscriptions have shown a satisfactory increase for some years, and it is expected that £4,000 will be reached by the end of the year. Yet the proportion contributed by limited liability companies and large firms is small, while the help rendered to injured workmen makes a duty to the institution imperative and clear. The result of the nine months' working has been satisfactory, and the board welcomed the willingness shown to support the great work of the infirmary—a willingness in which the workpeople of the town and county had taken no less an interest than those who had largely helped by capital sums. The report was adopted unanimously.

**West Bromwich District Hospital.**

The Mayor of West Bromwich must have been pleased at the ready response that was made by the borough to his appeal for one million pennies to perpetuate the memory of the late King, for the public meeting held in the Town Hall last week was quite a success. He went on to say that if it were possible for the late King to speak to them he would, he believed, express his approval of the scheme they were about to adopt, for their departed Monarch had always the keenest sympathy with hospitals. The President of the hospital (Edward F. Chance, Esq.) said that they were under a deep debt of gratitude to the Mayor for the task he was now undertaking, and he should be pleased to help in the effort by giving a donation of £200. The following gentlemen were appointed a Committee to assist the Mayor in carrying out the work, viz: Mr. E. F. Chance, Mr. F. T. Jefferson, Mr. J. G. Gifford, Mr. J. A. Kenrick, Mr. T. Foley Bache, Mr. S. Allsopp, Mr. James Clark, Mr. F. B. Barclay, Mr. A. H. Johnson, Mr. J. Lawley, Mr. Thos. Jones, Col. Walker, Alderman J. E. Wilson, and Dr. Plummer. Mr. H. M. Gunning (Lloyds Bank) and Mr. F. I. Hancock (Secretary of the Hospital) were appointed Hon. Treasurer and Hon. Secretary respectively. Before the close of the meeting the promises made reached the total of £767. This is a good beginning, and if the energy of the promoters of the scheme are kept up, there is little doubt that the money needed will be raised. It is a matter of experience that the raising of money is successful just in proportion to the efforts made to get it. No precise information has reached us as to whether payment of debt, improvement or extension is the aim for which the hospital will use the money.

**Norfolk and Norwich Hospital.**

The recent quarterly meeting of the governors of the Norfolk and Norwich Hospital had the satisfaction of hearing that a letter has been received from his Majesty giving permission to name the new septic and isolation block after King Edward VII., and it is hoped that an announcement will be made shortly of the date on which this notable addition will be opened formally. As regards the work of the past quarter, the report states that the daily average number of in-patients has been 172, while 2,648 persons have been treated at the out-patient department. Legacies amounting roughly to £1,601 have been received. Archdeacon Pelham, the chairman of the board of management, announced that a committee has been appointed to superintend the building of the new Eye Infirmary and of the theatres, and it is hoped that the buildings themselves will be ready for occupation by the beginning of the year. At the present moment the plans for the Eye Infirmary are being prepared for the scrutiny of the Charity Commissioners, the approval of whom will enable the building committee to start work, with the consent of the board of management. Another speaker was Dr. Barton, who defended the newly created appointment of honorary pathologist. The chairman of the House Committee then proposed the expenditure of £200, which was agreed to eventually, to provide eight beds for patients undergoing open-air treatment. Mr. Horsfall then went on to explain that the advantages of such treatment were not experienced only by consumptives, and it was with a view to extending its benefits on a smaller scale to patients generally that he urged the meeting to sanction the experiment. His proposal had the support of the medical staff.

**Orthopædic Hospital, Birmingham.**

The annual meeting of the Birmingham Orthopædic and Spinal Hospital had an interest of its own, for the very indebtedness revealed in the accounts points to the increase in the work done, and therefore provides the most direct proof of the inadequacy of the existing buildings, which has been complained of frequently. The operative work has increased by 50 per cent., and, it must be remembered, has to be performed in a building which, as the workers have to confess, "can recommend itself to no one for the purpose." In spite of the gentle pessimism of the Marquis of Hertford, who presided, we believe that if these facts were collated, and presented readably for Birmingham to grasp, money for a new building might be forthcoming. Appeals succeed when brains and active propaganda are found to back them, no matter how unfavourable circumstances may be. The success of the recent Middlesex appeal is an admirable example, for no worse year, apparently, than 1910 could have been found. The Orthopædic Hospital's annual expenditure for the past year was £3,124, that is to say, £1,032 more than its income. This, the inadequacy of the present building, and finally the increased work which has led to this indebtedness are facts which, we believe, might be used to illustrate each other in a way that would prove irresistible.

### THE MEDICAL SCHOOLS.

The following are next week's arrangements at the Medical Graduates' College and Polyclinic, 22 Chenies Street, W.C.: Monday, at 4 p.m., Dr. James Galloway, Clinique (skin); at 5.15 p.m., Dr. Chas. R. Box, "Syphilitic Hemiplegia in Childhood." Tuesday, at 4 p.m., Dr. Eric Pritchard, Clinique (medical); at 5.15 p.m., Dr. G. E. Herman, "Abortion." Wednesday, at 4 p.m., Mr. Charles Ryall, Clinique (surgical); at 5.15 p.m., Dr. G. H. Savage, "Recoveries, Remissions, and Relapses in Mental Disorder." Thursday, at 4 p.m., Mr. J. Thomson Walker, Clinique (surgical); at 5.15 p.m., Dr. William Pasteur, "Radiography in Medical Diagnosis." Friday, at 4 p.m., Mr. Sydney Stephenson, Clinique (eye).

The following are the arrangements for next week at the West London Post-graduate College: October 31 and November 3, at 10 a.m., demonstration by surgical registrar; October 31, at 12 noon, pathological demonstration; November 1, at 11.30 a.m., demonstration of minor operations; November 2, at 12.15 p.m., lecture, Dr. Grainger Stewart, practical medicine; November 4, at 10 a.m., demonstration by medical registrar. Lectures at 5 p.m.: October 31, Mr. Edwards, on Rectal Surgery; November 1, Dr. Davis, on Diseases of the Throat and Nose, the lines on which treatment should be based (Lecture II.); November 2, Dr. Beddard, on Practical Medicine (Lecture III.); November 3, Mr. Baldwin, on Practical Surgery; and November 4, Dr. Seymour Taylor, on "A Paralytic Stroke."

THE HOSPITAL October 29, 1910.

